# Supplementary material for: A set of multi-entry identification keys to African frugivorous flies (Diptera, Tephritidae)
Source: Zookeys. 2014 Jul 24;(428):97–108. doi: 10.3897/zookeys.428.7366 (PMC4143993; doi:10.3897/zookeys.428.7366)
Supplement: Supplementary material 4 — Key to Capparimyia [file zookeys-428-097-s004.zip › SF4_ZooKeys_key to Capparimyia/lucid_player/help/help/shortcuts_to_an_answer_-_the_shortcut_function.htm]

Shortcuts to an answer - The Shortcut function


# Shortcuts to an answer - The Shortcut function

The *Shortcuts* function is similar
to the *Best* functions in that
it may provide a quicker route to an identification. In *Shortcuts*
the Player searches for features that, if answered, would provide an immediate
shortcut to an identification.

For example, consider a key to fish. After choosing a number of states,
the list of *Entities Remaining*
may have been reduced to 20 species. Some of those species may have unique
features, or share a state with only one or two other species in the list.
If you were to choose such a state, you would immediately shortcut to
a single species or a very small number of species.

Shortcuts provides a way of finding such unique features. Find shortcuts
by clicking the *View Shortcuts*
button  on the button bar, or choosing *Shortcuts*
from the *Features* menu.

A list of shortcut features will be opened. Scan the list for features
that match your specimen (of course, there may be no available shortcut,
in which case the *Shortcuts* list
will be empty or the specimen being identified will not match any listed
feature). You may choose shortcut features by clicking on their score
boxes in the *Shortcuts* dialog.

**Note** The *Shortcuts*
function is similar to the *Bingo*
function in previous editions of the Lucid Player.
